# Functional Characterization of Human CYP2C9 Allelic Variants in COS-7 Cells

**DOI:** 10.3389/fphar.2016.00098

**Published:** 2016-04-25

**Authors:** Huihui Du, Zhiyun Wei, Yucai Yan, Yuyu Xiong, Xiaoqing Zhang, Lu Shen, Yunfeng Ruan, Xi Wu, Qingqing Xu, Lin He, Shengying Qin

**Affiliations:** ^1^Bio-X Institutes, Shanghai Jiao Tong UniversityShanghai, China; ^2^Shanghai Genome Pilot Institutes for Genomics and Human HealthShanghai, China; ^3^Department of Pharmacy, Shanghai Pulmonary HospitalShanghai, China; ^4^Institutes of Biomedical Sciences, Fudan UniversityShanghai, China

**Keywords:** cytochrome P450, genetic polymorphisms, pharmacokinetics, *in vitro* models, HPLC (high-performance/pressure liquid chromatography), S-warfarin, Chinese Han

## Abstract

Variability in activity of CYP2C9, which is involved in the metabolism of approximately 15% of current therapeutic drugs, is an important contributor to interindividual differences in drug response. To evaluate the functional alternations of CYP2C9^*^2, CYP2C9^*^3, CYP2C9^*^8, CYP2C9^*^11 and CYP2C9^*^31, identified in our previous study in Chinese Han population, allelic variants as well as the wild-type CYP2C9 were transiently expressed in COS-7 cells. Kinetic parameters (Km, Vmax, and Clint) for S-warfarin 7-hydroxylation by these recombinant CYP2C9s were determined. Relative to CYP2C9.1, recombinant CYP2C9.3 and CYP2C9.11 exhibited significantly higher Km values, and all allelic variants showed significantly decreased Vmax and Clint values. Among all allelic variants, catalytic activity of CYP2C9.3 and CYP2C9.11 reduced the most (8.2% and 9.8% of Clint ratio, respectively; *P* < 0.001). These findings should be useful for predicting the phenotype profiles of CYP2C9 in Chinese Han population, comparing the functional results of these alleles accurately, and finally optimizing pharmacotherapy of drug treatment.

## Introduction

Human cytochrome P450 2C9 (CYP2C9) is one of the most important members of the cytochrome P450 superfamily, accounting for ~20% of hepatic total CYP content and involved in the metabolism of approximately 15% of current therapeutic drugs, including antibiotic, anticancer, antidiabetic, antiepileptic, antihypertensive, cannabinol, non-steroidal anti-inflammatory, anticoagulant, and anti-hyperlipidemic drugs (Zhou et al., 2010). in addition, lots of endogenous compounds, such as progesterone, testosterone and arachidonic acid, are also metabolized by CYP2C9 (Rifkind et al., 1995; Yamazaki and Shimada, 1997). Alternation of CYP2C9 activity is an important contributor to the interindividual differences in drug response. For example, patients carrying CYP2C9^*^2 or CYP2C9^*^3, both of which will lead to reduced enzymatic activity, were reported to have higher risk for adverse drug responses, when they were treated with warfarin or S-acenocoumarol (Higashi et al., 2002; Malhi et al., 2004; Visser et al., 2004).

To date, 67 variants of *CYP2C9* (^*^1A through to ^*^60) located in the coding region have been identified (http://www.cypalleles.ki.se/cyp2c9.htm). Besides, different ethnic populations often show considerable differences in frequencies of CYP2C9 alleles. For example, CYP2C9^*^2 and CYP2C9^*^3 were reported to have relatively higher frequencies in Caucasians than in African-Americans, while CYP2C9^*^5 was found almost exclusively in African-Americans (Zhou et al., 2010). Consequently, functional characterization of different CYP2C9 variants, especially variants identified in the same ethnic population, is of great importance for the optimal pharmacotherapy of drug treatment, particularly for the appropriate dosing of drugs with a narrow therapeutic index such as warfarin.

In our previous study, we identified five CYP2C9 alleles in four geographically different Chinese Han populations, namely CYP2C9^*^2, CYP2C9^*^3, CYP2C9^*^8, CYP2C9^*^11, and CYP2C9^*^31 (Xiong et al., 2011). Even though there have been many reports concerning functional characterization of these CYP2C9 allelic variants *in vitro*, some of these studies reported discordant kinetic parameters of the same CYP2C9 variant toward the same substrate. For example, Kaminsky et al. (1993) reported that yeast-expressed CYP2C9.2 exhibited increased catalytic activity toward S-warfarin hydroxylation, while Rettie et al. (1994, 1999) reported that recombinant CYP2C9.2 expressed in insect cells or HepG2 cells showed decreased activity toward S-warfarin metabolism. Besides, most of these studies were performed using recombinant CYP2C9s expressed in *E.coli*, yeast or insect cells (Hiratsuka, 2012), which were less relevant with human CYP2C9 variant proteins than mammalian cell-expressed CYP2C9s. What is more, most of previous studies using the mammalian cell-based expression system only focused on parts of these CYP2C9 alleles (Hiratsuka, 2012). It is difficult to assess activity alternation of these CYP2C9s with data obtained under different experiment conditions. To our knowledge, only Niinuma et al. (2014) have studied all of these alleles with the mammalian cell based expression system. However, they obtained only parts of accurate kinetic parameters of all alleles, which is not enough to compare the activity alternation comprehensively.

To get a systematical and accurate assessment on the functional alternations of these CYP2C9 alleles, which is useful for predicting the CYP2C9 phenotype profile and optimizing drug administration in Chinese Han population, we characterized their enzymatic activity *in vitro* using S-warfarin as a representative substrate. Recombinant CYP2C9s were prepared by transfecting COS-7 cells with cDNAs of CYP2C9^*^1, CYP2C9^*^2, CYP2C9^*^3, CYP2C9^*^8, CYP2C9^*^11, and CYP2C9^*^31. Enzymatic activity of them toward S-warfarin 7-hydroxylation were examined by HPLC (High Performance Liquid Chromatography).

## Materials and methods

### Construction of expression plasmids

CYP2C9^*^1 cDNA in pCMV6-XL5 plasmid (Origene, Rockville, MD, USA) was released by digestion with *EcoR I* and *Xba I* (Thermo Scientific, Beijing, China), and then subcloned into pcDNA3.1 (±) vector (Invitrogen, Carlsbad, CA, USA). Site-directed mutagenesis to introduce the 430C>T (CYP2C9^*^2), 449G>A (CYP2C9^*^8), 980T>C (CYP2C9^*^31), 1003C>T (CYP2C9^*^11), and 1075A>C (CYP2C9^*^3) transitions was performed using pcDNA3.1 (±) carrying human CYP2C9^*^1 cDNA as the template for polymerase chain reaction amplification by *Pyrobest* DNA polymerase (TaKaRa, Dalian, China). The specific base transition was introduced into the amplification products by a pair of completely complementary primers containing the substituted base (Supplemental Table 1). After incubation with *Dpn I* (Thermo Scientific) to digest the template and purification, these vectors were transformed into *E. coli* Top 10 (Tiangen, Beijing, China), and then purified with NucleoBond Xtra plasmid purification Kit (MACHEREY-NAGEL, Germany). Clones carrying the desired mutants were identified by direct DNA sequencing. DNA concentration and quality were evaluated with Nano Drop 2000 UV-Vis Spectrophotometer (Thermo, Wilmington, DE, USA).

## Transfection of COS-7 cells and preparation of microsomes

COS-7 cells were seeded on 10-cm culture dishes in Dulbecco's modified Eagle's medium (DMEM; ATCC, VA, USA) containing 10% Fetal bovine serum (FBS; PAA, Piscataway, NJ, USA), 100 U/mL penicillin (Invitrogen), 100 U/mL streptomycin (Invitrogen), and 0.01 mg/mL Plasmocin (Invitrogen). When cells were ~90% confluent, vectors carrying desired cDNAs were transfected into the COS-7 cells with TransFectin Lipid Reagent (Bio-Rad, Hercules, CA, USA) according to the manufacturer's guidelines. The optimal transfection efficiency and cell viability were obtained with 24 μg DNA/dish and 50 μL TransFectin Lipid Reagent. 48 h after transfection, cells were scrapped from the culture dishes, washed twice with 100 mM potassium phosphate buffer (pH 7.4), and then resuspended in 20 mM potassium phosphate buffer (pH 7.4), containing 0.2 mM EDTA, 1 mM dithiothreitol, and 20% glycerol (Guo et al., 2005). After sonication, the homogenate was centrifuged at 9000 g, 4°C for 20 min. Subsequently, the resulting supernatant was centrifuged at 105,000 g, 4°C for 60 min. The microsomal pellet was resuspended in 250mM sucrose and stored at −80°C.

## Determination of COS-7 expressed CYP2C9 variant proteins

Total protein concentration of COS-7 cell expressed microsomes was measured by Bradford method (Bradford, 1976) using the Bio-Rad protein assay (Bio-Rad) according to the manufacturer's guidelines. Microsomal CYP2C9 apoprotein levels were determined by immunoblotting, with a serial of dilution of baculovirus-expressed CYP2C9 (BD, San Jose, CA, USA) as the standard. Recombinant proteins were separated on 10% sodium dodecyl sulfate-polyacrylamide gels and transferred to polyvinylidene difluoride membranes (Millipore Corporation, Billerica, MA). After incubation with the rabbit-produced anti-CYP2C9 antibody (Sigma-Aldrich, St. Louis, MO, USA), the primary antibody, at 4°C for 24 h, the membrane was incubated with the HRP-conjugated anti-rabbit IgG (Sigma-Aldrich) for 2 h at room temperature. Bands were visualized by incubating the membrane with the chemiluminescent peroxidase substrate (Sigma-Aldrich), and analyzed with Image J software (National Institutes of Health, Bethesda, MD, USA). Experiments were conducted in triplicate independently.

## *In vitro* S-warfarin 7-hydroxylation and HPLC analysis

For the preparation of the (S)-warfarin stock solution [1 mM (S)-warfarin in 5 mM KOH], 0.0145 mmol (S)-warfarin was dissolved in 70 ul 1 M KOH and further diluted with water to a final volume of 14.5 ml. Each incubation mixture of (S)-warfarin contained a final concentration of 0.5 mM KOH; the final pH of the incubation mixtures was 7.4 (Hemeryck et al., 1999).

S-warfarin (2–100 μM; Sigma-Aldrich) was incubated at 37°C with microsomes(5–20 pmol) expressed from COS-7 cells and a NADPH-generating system (consisting of 1 mM NADP^+^, 10 mM glucose-6-phosphate, 5 mM MgCl_2_ and 2 U/mL glucose-6-phosphate dehydrogenase; Sigma-Aldrich) in 0.1 M potassium phosphate buffer (pH 7.4). The total volume of every incubation was 250 μL. Reactions were initiated by adding the NADPH-generating system, after the pre-incubation (37°C, 5 min). Then after 2-h incubation with gentle shaking, reactions were quenched with 100 μL cold acetonitrile, containing 0.2 μg 7-Ethoxycoumarin (Sigma-Aldrich) as the internal standard. Subsequently, the mixture was centrifuged at 12,000 g, 4°C for 20 min. Resulting supernatant was then passed through a syringe nylon filter (13 mm, 0.45 μm; Dikma Technologies, Lake Forest, CA, USA), and 50 μL of the filtrate was injected for HPLC analysis. Every reaction was performed in triplicate independently.

HPLC analysis was performed using the Agilent 1260 HPLC system. 7-Hydroxywarfarin (Sigma-Aldrich) and the internal standard 7-ethoxycoumarin were separated on an Agilent ZORBAX SB-C18 column at ambient temperature. The mobile phase, a mixture of acetonitrile and 0.5% phosphoric acid (38:62, V/V), was pumped at a flow rate of 1 mL/min. The lower limit of 7-hydroxywarfarin quantification was 0.20 nM. Fluorescence detection was performed at an excitation wavelength of 320 nm and an emission wavelength of 415 nm (Lang and Bocker, 1995). Under these conditions, retention time of 7-hydroxywarfarin and 7-ethoxycoumarin was 7.3 and 8.7 min, respectively. A 7-point standard curve was used to quantify the amount of 7-hydroxywarfarin in samples.

## Data analysis

Brands of Western Blot were analyzed using Image J software. The standard curve of baculovirus-expressed CYP2C9.1 and that of the 7-hydroxywarfarin were made using OriginPro 7.5 (OriginLab, Northampton, MA, USA) with linear fitting. Prism 4.0 software (GraphPad, San Diego, CA, USA) was used to calculate kinetic parameters of different CYP2C9 variants toward S-warfarin 7-hydroxylation with nonlinear regression analysis of a Michaelis-Menten equation. Statistical comparisons were performed using IBM SPSS Statistics 14.0 (IBM, Armonk, NY, USA) with Dunnett *t*-tests, which treat one group as a control, and compare all other groups against it. It was considered to be statistically significant when the value of *P* < 0.05.

## Results

**Figure 1:**
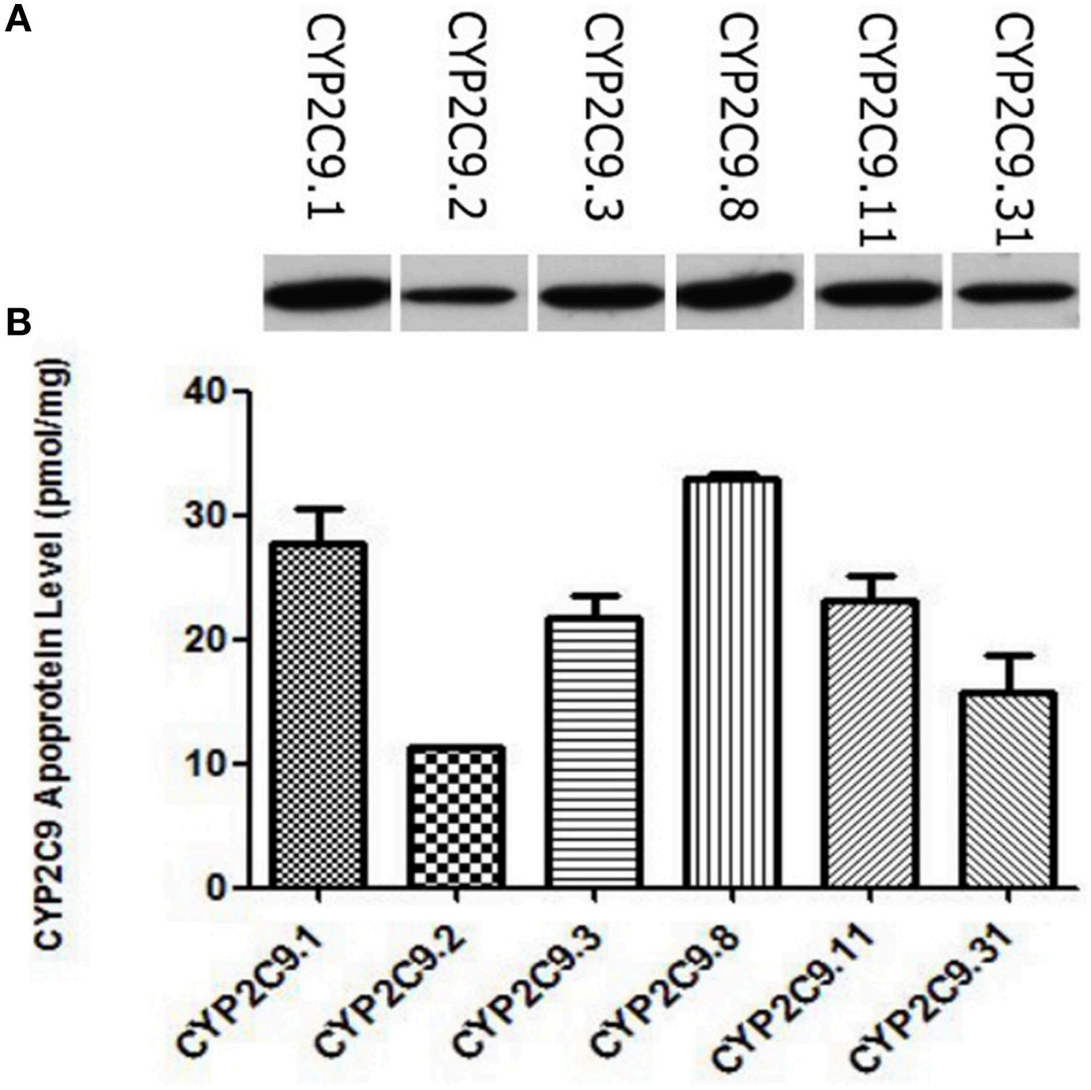
**Detection of CYP2C9 proteins by western-blot analysis (A) and the relative expression level of CYP2C9 proteins expressed in COS-7 cells (B)**. Recombinant CYP2C9s were recognized by rabbit-produced anti-CYP2C9 antibody, and experiments were conducted in triplicate independently.

The wild type CYP2C9 and these five allelic variants identified in our previous study were expressed in COS-7 cells. Apoprotein levels of these recombinant CYP2C9s were measured by immunoblotting with baculovirus-expressed CYP2C9 as the standard. As shown in Figure 1A, all of them were immunodetectable with the rabbit-produced anti-CYP2C9 antibody. Using brands of baculovirus-expressed CYP2C9.1 with known quantities as the standard curve, the CYP2C9 apoprotein contents in COS-7 cell-generated microsomes could be quantified (Figure 1B). The expression level of CYP2C9.1 was 27.75 ± 2.73 (mean ± S.D.) pmol/mg microsomal protein. Statistical comparisons (Dunnett *t*-tests) between CYP2C9.1 (the wild type of CYP2C9) and other CYP2C9 allelic variants showed that the expression levels of CYP2C9.3, CYP2C9.8, CYP2C9.11 had no statistical difference (*P* = 0.05) from that of CYP2C9.1. The expression level of CYP2C9.2 was the lowest, with an amount of 11.24 ± 0.16 (mean ± SD) pmol/mg microsomal protein (40.5% of CYP2C9.1 expression level, *P* < 0.001). CYP2C9.31 exhibited moderate decreased expression level (56.6% of that of CYP2C9.1, *P* < 0.05).

**Figure 2:**
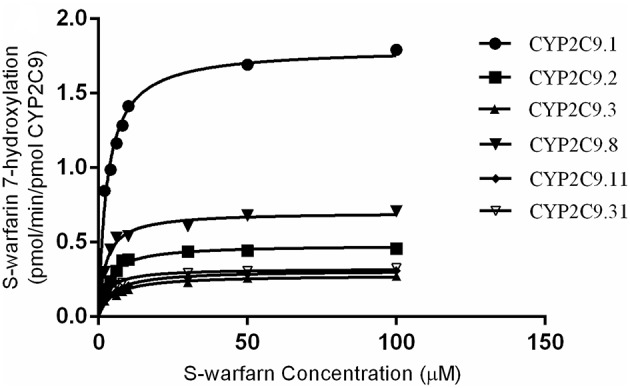
**Kinetics of S-warfarin 7-hydroxylation by COS-7 cell-expressed CYP2C9.1, CYP2C9.2, CYP2C9.3, CYP2C9.8, CYP2C9.11 and CYP2C9.31**. Every point represents the mean of three independent experiments.

*In vitro* catalytic activity of these COS-7 cell-expressed CYP2C9 toward S-warfarin 7-hydroxylation was evaluated with experiments conducted in triplicate independently. As shown in Figure 2, all recombinant CYP2C9s presented typical Michaelis-Menten kinetic profiles. Kinetic parameters (*K*_*m*_, *V*_*max*_ and *CL*_*int*_) of them were summarized in Table 1, with data shown in the form of mean ± S.D. The estimated kinetic parameters *K*_*m*_, *V*_*max*_ and *Cl*_*int*_ of COS-7 cell-expressed CYP2C9.1 (wild type) toward S-warfarin 7-hydroxylation were 2.92 μM, 1.80 pmol/min/pmol CYP2C9 and 0.61 μL/min/pmol CYP2C9, respectively. Relative to the wild type CYP2C9, recombinant CYP2C9.3 and CYP2C9.11 exhibited significantly higher *K*_*m*_ values (1.64-fold and 1.52-fold, respectively; *P* < 0.001). Besides, all variants showed significantly lower *V*_*max*_(*P* < 0.001). Among them, the *V*_*max*_ value for CYP2C9.3 is the lowest, which was only 15% of the wild type. Accordingly, all recombinant variants exhibited significantly decreased *Cl*_*int*_ values (*P* < 0.001), among which *Cl*_*int*_ values for CYP2C9.3 and CYP2C9.11 decreased the most (8.2% and 9.8% of CYP2C9.1, respectively).

**Table 1:** ***In vitro* kinetic parameters for S-warfarin 7-hydroxylation by COS-7 cell-expressed CYP2C9.1, CYP2C9.2, CYP2C9.3, CYP2C9.8, CYP2C9.11, and CYP2C9.31**.

**Variant**	**Nucleotide transitions**	***K*_*m*^a^_**	***V*_*max*^a^_**	***CL*_*int*^a^_**
		**μM**	**pmol/min/pmol CYP2C9**	**μL/min/pmol CYP2C9**
CYP2C9.1	Wild type	2.92 ± 0.25	1.80 ± 0.040	0.61 ± 0.039
CYP2C9.2	430 C>T	3.13 ± 0.23	0.48 ± 0.005^***b^	0.15 ± 0.010^***b^
CYP2C9.3	1075 A>C	4.78 ± 0.10^***b^	0.27 ± 0.001^***b^	0.05 ± 0.000^***b^
CYP2C9.8	449 G>A	2.43 ± 0.24	0.70 ± 0.014^***b^	0.28 ± 0.023^***b^
CYP2C9.11	1003 C>T	4.44 ± 0.04^***b^	0.31 ± 0.001^***b^	0.06 ± 0.000^***b^
CYP2C9.31	980 T>C	2.66 ± 0.14	0.32 ± 0.002^***b^	0.12 ± 0.005^***b^

a Each value represents the mean ± SD of three independent experiments.

b*** P < 0.001 vs. wild type with a Dunnett t-test.

## Discussion

Variability in CYP2C9 activity is an important contributor to interindividual drug response variations, especially for drugs with a narrow therapeutic index, such as Warfarin. In the present study, we focused on five CYP2C9 alleles (CYP2C9^*^2, CYP2C9^*^3, CYP2C9^*^8, CYP2C9^*^11, and CYP2C9^*^31) identified in Chinese Han population, expressed all of them as well as the wild type enzyme in COS-7 cells, and characterized their catalytic activity with S-warfarin as the representative substrate. Apoprotein levels of these recombinant CYP2C9s were measured by immunoblot analysis, because accurate quantification of their holoprotein levels by CO difference spectroscopy was reported to be difficult due to low levels of expression (Hiratsuka, 2012). Even though immunoblot analysis might overestimate the P450 levels of these CYP2C9 levels, resulting in the overestimation of *Cl*_*int*_ values, this method has gained its popularity in estimating the apoprotein levels of CTP2C9 as well as many other CYPs, including CYP2D6, CYP2B6, and CYP2E1, especially when mammalian cells were used as the expression system (Marcucci et al., 2002; Hanioka et al., 2003; Watanabe et al., 2010).

Catalytic activity of these 5 CYP2C9 variants toward S-warfarin 7-hydroxylation was measured with triple independent experiments. Compared with the wide type, all allelic variants exhibited impaired enzymatic activity. Among them, *in vitro* functional characterization of CYP2C9^*^2 and CYP2C9^*^3 have been reported most extensively. In most of the previous studies, both CYP2C9^*^2 and CYP2C9^*^3 were associated with impaired catalytic activities, such as lower *V*_*max*_ and higher intrinsic clearance values (Rettie et al., 1994), higher *K*_*m*_ and lower *Cl*_*int*_ values (Guo et al., 2005), toward a variety of CYP2C9 substrates, relative to the wild type. In our present study, both recombinant CYP2C9.2 and CYP2C9.3 exhibited impaired catalytic activity toward S-warfarin 7-hydroxylation (24.6% and 8.2% of *Cl*_*int*_ ratio, respectively), which were similar to values reported previously. The mechanisms involved in the decreased catalytic activities of CYP2C9.2 and CP2C9.3 may be related to the enzyme conformational changes. Wei et al. (2007) reported that the conversion of enzyme to the high spin state was similar in CYP2C9.1 and CYP2C9.2, but lower in CYP2C9.3; and neither altered substrate binding nor altered interaction with reductase appeared to be involved in the reduced catalysis. Besides, the R144C substation caused by CYP2C9^*^2 was suggested to affect the interaction between CYP2C9 enzyme and the P450 reductase, while the I359L substitution caused by CYP2C9^*^3 might affect the substrate recognition (Gotoh, 1992; Crespi and Miller, 1997).

The CYP2C9^*^8 (449G>A) allele resulted in an R150H substitution with a frequency of 1.8% in Chinese Han populations (Xiong et al., 2011). In the present study, the COS-7 cell-expressed CYP2C9.8 exhibited a reduced value of intrinsic clearance (45.9% of the wild type) and a significantly lower *V*_*max*_ value (38.9% of the wild type) toward S-warfarin 7-hydroxylation. Our results are in accordance with the previously reported studies both *in vivo* and *in vitro*. Recently, Liu et al. (2012) reported that patients carrying the CYP2C9^*^8 allele exhibited a 30% reduction in the unbound oral clearance of S-warfarin than CYP2C9^*^1 homozygotes; and the *in vitro* intrinsic clearance of cDNA-expressed CYP2C9.8 toward S-warfarin metabolism was also 30% lower as compared with the wild type protein. Along with previous studies, our findings might provide explanations for the lower warfarin dose requirements in patients with the CYP2C9^*^8 allele.

CYP2C9^*^11 is a C1003T mutation in exon 7, leading to an Arg335Trp substitution. It has been detected in Caucasians, Africans as well as Asians (Allabi et al., 2005; Xiong et al., 2011). The Arg335 is located in the turn between the J and J' helices and forms a hydrogen-bonding ion pair with Asp341 from the J' helix, which plays an important role in the secondary structure stabilization. Blaisdell et al. (2004) Thus the Arg335Trp substitution will abolish this interaction and alter the substrate affinity. In our study, the COS-7 cell-expressed CYP2C9.11 showed a 1.5-fold higher *K*_*m*_ value, significantly lower *V*_*max*_ and reduced intrinsic clearance (*P* < 0.001) than the wild type. In the most recent report of Niinuma et al. (2014), CYP2C9.11 exhibited significantly lower catalytic activity for 40 μM S-warfarin than wild-type CYP2C9.1, although they did not determine the Michaelis–Menten kinetics for CYP2C9.11. Besides, Blaisdell et al. (2004) reported that the *E. coli*-expressed CYP2C9.11 exhibited a three-fold increase in the *K*_*m*_ and more than two-fold decrease in the intrinsic clearance for tolbutamide; and Allabi et al. (2005) reported that, in black population, there was a marked reduction of CYP2C9 activity in subjects carrying at least one CYP2C9^*^11 allele. Our findings are also in line with both *in vitro* and *in vivo* studies previously reported.

The CYP2C9^*^31 allele was detected in Africans firstly, and was predicted to have a possible functional damage (Matimba et al., 2009). Although there have been no reports about *in vivo* functional assessment of CYP2C9^*^31 by now, Niinuma et al. (2014) reported that the COS-7 cell-expressed CYP2C9.31 exhibited significantly decreased enzyme activity, when using S-warfarin as the representative substrate, without determining the kinetic parameters (*K*_*m*_, *V*_*max*_ and *Cl*_*int*_). Dai et al. (2013) reported that the insect cell-expressed CYP2C9.31 showed significantly reduced intrinsic clearance value (34.4% of the wild type). Ji et al. (2015) reported that the insect cell-expressed CYP2C9.31 exhibited significantly lower intrinsic clearance value than CYP2C9^*^1 toward fluoxetine. However, in contrast to their previous *in vitro* metabolic assessments on tolbutamide, losartan and glimepiride, CYP2C9.31 exhibited substrate inhibition trend toward fluoxetine, which did not follow the Michaelis–Menten kinetics. In our findings, the estimated *K*_*m*_, *V*_*max*_ and *Cl*_*int*_ values for CYP2C9.31 toward S-warfarin 7-hydroxylation were 2.66 μM (not significantly different from CYP2C9.1), 0.32 pmol/min/pmol CYP2C9 (17.8% of the wild type, *P* < 0.001) and 0.12 μL/min/pmol (19.7% of the wild type, *P* < 0.001), respectively. All these findings suggested that this allele was associated with significantly decreased enzymatic activity and patients carrying this allele should be given different drug treatments from patients carrying the wild type CYP2C9.

In conclusion, we expressed these five CYP2C9 allelic variants as well as the wild type CYP2C9 in COS-7 cells. The Michaelis–Menten kinetics for all these recombinant CYP2C9s were determined with S-warfarin as the representative substrate. All allelic variants exhibited significantly decreased catalytic activity. In the present study, we have not given the results of the combined CYP2C9 variants for their low minor allele frequencies (MAF) based on our previous study (Xiong et al., 2011). However, it is still possible that haplotypes may give different results. These findings should be useful for predicting the phenotype profiles of CYP2C9 in Chinese Han population, which could contribute to optimizing dosage recommendations, maximizing the efficacy and minimizing the adverse responses of drug treatment, particularly for Chinese Han population.

## Author contributions

HD and ZW did the experiments and completed this manuscript. YY and YX provided assistance on processing of the data. LS, YR, and XW provided assistance on the collection of the materials. QX helped in processing the figures. LH helped revise this manuscript. SQ, the Corresponding Author, designed and revised this manuscript.

### Conflict of interest statement

The authors declare that the research was conducted in the absence of any commercial or financial relationships that could be construed as a potential conflict of interest.
