# Supplementary material for: Functional Characterization of Human CYP2C9 Allelic Variants in COS-7 Cells
Source: Front Pharmacol. 2016 Apr 25;7:98. doi: 10.3389/fphar.2016.00098 (PMC4843492; doi:10.3389/fphar.2016.00098)
Supplement: Supplementary file 1 [file Table1.doc]

Supplemental materials

Functional Characterisation of Human CYP2C9 Allelic Variants in COS-7 cells

Huihui Du^1, 2, &^, Zhiyun Wei^1, 2, &^, Yucai Yan^1, 2^, Yuyu Xiong^1, 2^, Xiaoqing Zhang^3^, Lu Shen^1, 2^, Yunfeng Ruan^1, 2^, Xi Wu^1, 2^, Qingqing Xu^1, 2^, Lin He^1, 2, 4^, Shengying Qin^1, 2, *^

Supplemental Table 1 Primers used to introduce specific base transition

| *Allele* | Nucleotide transitions | Amino-acid changes | Primers |
| --- | --- | --- | --- |
| *CYP2C9*2* | 430 C>T | R144C | 5'-GAGGAGCATTGAGGACTGTGTTCAAGAGGAAGC-3' |
| *CYP2C9*3* | 1075 A>C | I359L | 5'-CGAGGTCCAGAGATACCTTGACCTTCTCCCCAC-3' |
| *CYP2C9*8* | 449 G>A | R150H | 5'-TCAAGAGGAAGCCCACTGCCTTGTGGAGG-3' |
| *CYP2C9*11* | 1003 C>T | R335W | 5'-CGTGTGATTGGCAGAAACTGGAGCCCCTGCA-3' |
| *CYP2C9*31* | 980 T>C | I327T | 5'-CACAGCTAAAGTCCAGGAAGAGACTGAACGTGTGAT-3' |

*^a.^* Mutations are underlined.
